# Long and Massive Thrombus in the Left Coronary Artery: A Case of Acute Myocardial Infarction Treated Using Immediate Stenting and Direct Oral Anticoagulant Plus Dual Antiplatelet Therapy

**DOI:** 10.1155/cric/1647428

**Published:** 2025-11-07

**Authors:** Nobuhiro Honda, Keita Inanaga, Jun-ichiro Nishi, Shujiro Inoue

**Affiliations:** Department of Cardiology, Aso Iizuka Hospital, Iizuka City, Fukuoka, Japan

**Keywords:** acute myocardial infarction, direct oral anticoagulant, dual antiplatelet agents, long and massive thrombus

## Abstract

**Background:**

Managing massive thrombi in the coronary arteries of patients with acute myocardial infarction presents considerable challenges, and the effectiveness of immediate versus deferred stenting as a treatment option remains uncertain. Moreover, it is unclear whether dual antiplatelet plus direct oral anticoagulant therapy is more effective for massive thrombi in the coronary arteries than dual antiplatelet therapy alone. We report the case of a patient with acute myocardial infarction with a long and massive thrombus in the left anterior descending artery treated with an immediate stenting strategy and antithrombotic combination therapy.

**Case Presentation:**

A 90-year-old female presented with a chief complaint of chest depression due to a long and massive thrombus in the left anterior descending artery with associated acute myocardial infarction. Immediate stenting to cover the ruptured plaque and trap the massive thrombus without encountering slow flow/no-reflow phenomenon, and employing thrombolysis through a combination of antiplatelet agents and a direct oral anticoagulant resulting in sustained coronary blood flow during the transition from the acute to subacute phases, led to favorable procedural and clinical outcomes.

**Conclusions:**

If immediate stenting with thrombolysis using a direct oral anticoagulant plus dual antiplatelet agents is successful without distal embolism, this strategy could be a better treatment than deferred stenting in terms of preventing periprocedural cardiac adverse events and reducing healthcare costs. Furthermore, dual antiplatelet agents plus direct oral anticoagulant combination therapy are considered to suppress platelet function and fibrin generation strongly, leading to the disappearance of thrombus.

## 1. Introduction

Intracoronary thrombosis in patients with acute myocardial infarction (AMI) can cause distal embolism, no-reflow phenomenon, stent thrombosis, and an increased risk of adverse cardiac events and death following primary percutaneous coronary intervention (PCI) [1, 2]. The debate continues on whether immediate or deferred stenting strategies for primary PCI are more effective for the procedural and clinical outcomes of massive intracoronary thrombi in patients with AMI [3–7]. Moreover, it is unclear whether a direct oral anticoagulant (DOAC) added to standard antiplatelet agents is effective for AMI with massive intracoronary thrombi.

Herein, we report a case of AMI with large thrombus-induced aspiration thrombectomy failure. Subsequent successful thrombolysis was achieved through immediate stenting for culprit lesion covering, thrombus trapping, and administering a DOAC alongside dual antiplatelet agents. Notably, this approach resulted in a procedure free of complications and yielded favorable clinical outcomes.

## 2. Case Presentation

**Figure 1:**
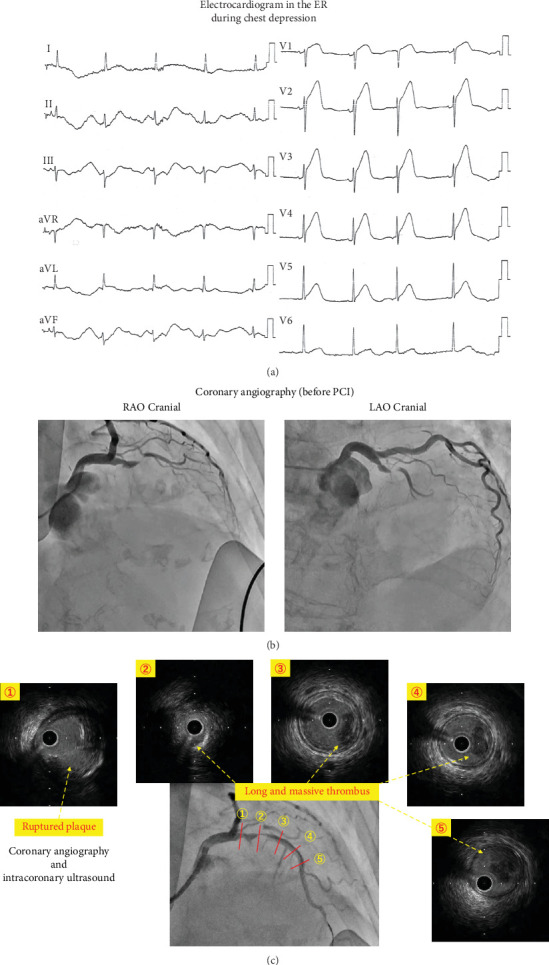
Electrocardiogram, coronary angiography, and intracoronary ultrasound. (a) Electrocardiography in the emergency room. Electrocardiogram showing evidence of ST-segment elevation in the precordial (V1–5) and lateral leads and the reciprocal change in the inferior leads. (b) Coronary angiography before percutaneous coronary intervention. Coronary angiography showing an occlusion in the left anterior descending artery in the proximal portion. (c) Coronary angiography and intravascular ultrasound during percutaneous coronary intervention. Coronary angiography and intravascular ultrasound showing a long and massive thrombus in the left anterior descending artery from the proximal to the middle portion.

A 90-year-old woman with chest depression was transported to our institution by ambulance. She took medications for hypertension and dyslipidemia, had never smoked, and had no family history of sudden death or cardiac arrest. Electrocardiography performed in the emergency room showed ST-segment elevation in the precordial (V1–5) and lateral leads and reciprocal change in the inferior leads (Figure 1a). Transthoracic echocardiography revealed severe hypokinesis in the basal and middle segments of the septal and apical walls. Her blood pressure and heart rate were 140/80 mmHg and 60 bpm, respectively, while her hemodynamic status was stable. She had a serum troponin I level of 164.5 pg/mL and was categorized into Killip Class 1. Her body mass index was 20.2. Oral aspirin (300 mg), prasugrel (20 mg), and intravenous unfractionated heparin (5000 IU) were administered in the emergency room. The patient was admitted to the catheterization laboratory with a diagnosis of AMI with ST-segment elevation. On admission, coronary angiography (CAG) was performed via the radial approach approximately 2 h after the onset of symptoms. This procedure revealed a left anterior descending artery (LAD) completely occluded proximally by a thrombus (Figure 1b). The coronary arteries showed no bilateral ectasia or structural anomalies.

**Figure 2:**
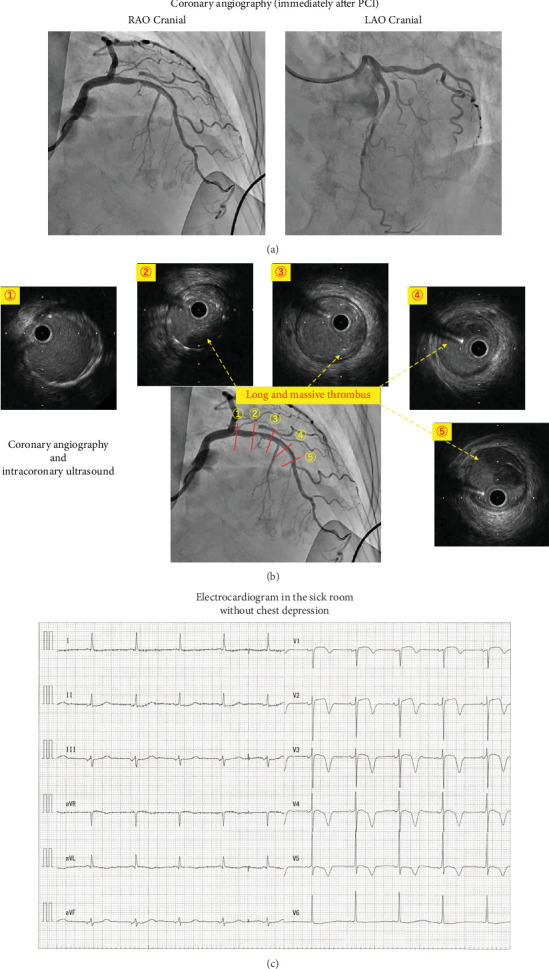
Coronary angiography, intravascular ultrasound, and electrocardiogram. (a) Coronary angiography after stenting. Coronary angiography showing improved coronary blood flow with a remaining long-filling defect in the left anterior descending artery. (b) Coronary angiography and intracoronary ultrasound after stenting. Coronary angiography and intracoronary ultrasound showing the stent covering the culprit lesion and trapping the thrombus. (c) Electrocardiogram in the ward. Electrocardiogram showing ST-segment complete resolution in precordial leads.

After administering another bolus of unfractionated heparin (5000 IU), manual aspiration thrombectomy was performed using a 7-Fr aspiration catheter (Rebirth Pro, Goodman Co. Ltd., Nagoya, Japan). Despite the inability to completely eliminate the thrombi, the intervention successfully led to the attainment of thrombolysis in myocardial infarction (TIMI) Grade 2 flow. A large filling defect persisted in the LAD from the proximal to the middle portion. Intravascular ultrasound (AltaView, Terumo, Tokyo, Japan) revealed a ruptured plaque with a large thrombus approximately 40 mm in length in the LAD (Figure 1c). Additional manual aspiration thrombectomy was performed; however, the large thrombus could not be aspirated. Considering the high bleeding risk due to the patient's advanced age, intracoronary thrombolytic therapy was not performed. Although the luminal diameter of the LAD was normal, this thrombus occupied one-fourth to one-third of its area without a culprit lesion. Furthermore, the luminal area around the culprit lesion was almost entirely occupied by fibrous lipid-rich plaques. Hence, the decision was made to proceed with immediate stenting solely for covering the ruptured plaque and effectively trapping the thrombus. A drug-eluting stent (Xience Skypoint 3.0/18 mm, Abbott, United States) was inserted into the culprit lesion in the proximal LAD. Because intravascular ultrasound revealed malapposition of the proximal edge of the stent, a 3.75 × 8 mm noncompliant balloon (Hiryu-Plus, Terumo, Tokyo, Japan) was inflated proximal to the stent for apposition. The final CAG showed improved coronary blood flow, from TIMI Grade 2 to almost 3, with a long filling defect remaining in the LAD (Figure 2a). The final intravascular ultrasound revealed stent coverage of the ruptured plaque with a persistent long and massive thrombus in the LAD (Figure 2b). The chest depression completely improved, and the ST segment elevation was resolved (Figure 2c). Hence, the primary PCI was completed.

**Figure 3:**
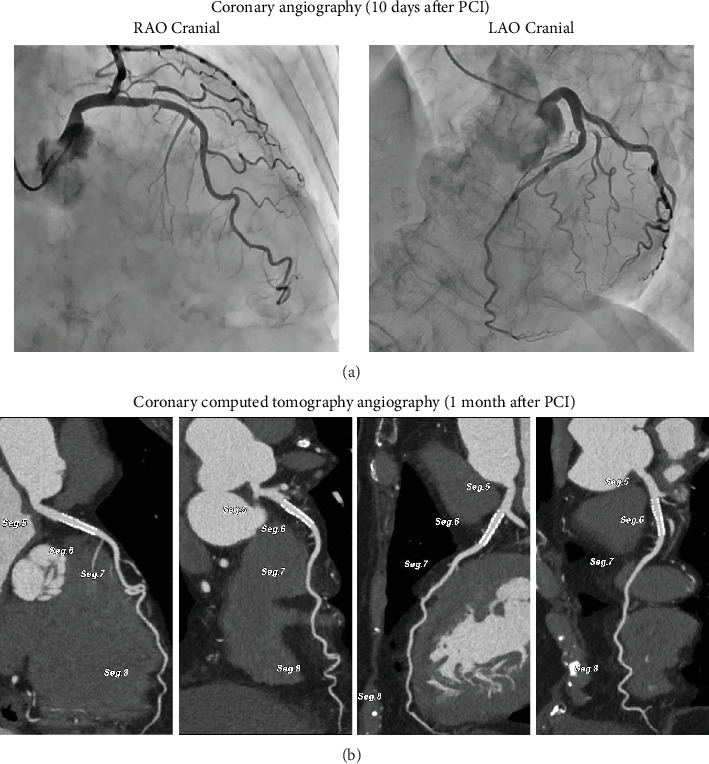
Coronary angiography and coronary computed tomography angiography. (a) Coronary angiography 10 days after percutaneous coronary intervention. Coronary angiography showing a tiny thrombus in the stent. (b) Coronary computed tomography angiography 1 month after the percutaneous coronary intervention. Coronary computed tomography angiography showing no thrombus in the left anterior descending artery.

Unfractionated heparin infusion was maintained until a day after the PCI. Rivaroxaban (10 mg/day), aspirin (100 mg/day), and prasugrel (3.75 mg/day) were orally administered from the day after the PCI. No significant bleeding or post-AMI complications occurred after PCI. Staged CAG repeated 10 days after PCI showed TIMI Grade 3 blood flow without a large thrombus in the LAD and with a small in-stent thrombus (Figure 3a). Based on these CAG findings, we stopped the DOAC treatment and administered dual antiplatelet agents continuously. The hemoglobin level was maintained at approximately 12–13 g/dL without blood transfusion during hospitalization. The patient was discharged 12 days after the PCI. Coronary computed tomography was performed 1 month after PCI and revealed no thrombus in the LAD (Figure 3b). Dual antiplatelet therapy was reduced to a single agent 6 months after the PCI. During 12 months of follow-up, the patient did not experience recurrent chest discomfort while using a single antiplatelet agent.

## 3. Discussion

We present a case of a large thrombus in the LAD with associated AMI. The presence of this large thrombus posed challenges during the primary PCI. Despite the difficulties encountered in managing the substantial thrombus, its incorporation into the culprit lesion was addressed, resulting in enhanced and sustained coronary blood flow during the transition from the acute to subacute phases. In this case, the patient was administered an oral DOAC and dual antiplatelet agents after the PCI. Dual antiplatelet agents plus the DOAC were considered to suppress platelet function and fibrin generation, leading to the disappearance of the thrombus. In vitro and in vivo studies suggested that combining rivaroxaban with dual antiplatelet agents worked synergistically to reduce platelet activation, leading to delayed or reduced formation of coagulation complexes, thereby enhancing antithrombotic potency [8]. This study also indicated that a dual-pathway approach with rivaroxaban and dual antiplatelet agents could synergistically improve antithrombotic therapy in AMI. In the present case, the large thrombus was reduced to a tiny in-stent thrombus on Day 10 and disappeared within 1 month of combination therapy following the dual therapy. Because the peak creatine kinase level was approximately 1400 IU/L, the MI damage was relatively small, and the combination of rivaroxaban and an antiplatelet agent strategy may be beneficial for massive thrombus in AMI.

The patient's advanced age made her highly susceptible to bleeding. Prior studies on dual antiplatelet therapy plus DOAC after PCI have shown that this combination therapy increases bleeding events [9–12]. Thus, this combination therapy has not been maintained for long, particularly in older adults. Moreover, these studies demonstrated that most bleeding events occur within 1 month of introducing antithrombotic agents after PCI. Therefore, if combination therapy is performed after PCI, it should be terminated as quickly as possible to avoid bleeding. Although we do not have sufficient evidence on the effectiveness of DOAC plus antiplatelet therapy for the treatment of AMI with massive thrombi or how the duration of this combination therapy is better for thrombolysis, DOAC plus dual antiplatelet therapy within 1 month was a useful treatment for AMI with massive thrombus in previous case reports [13, 14]. Therefore, we consider this strategy effective for treating AMI with massive thrombi within 1 month.

The effectiveness of immediate or deferred stenting strategies in the treatment of AMI with massive thrombi remains controversial [3–7]. Randomized controlled trials have shown no differences in the procedural and clinical outcomes [6, 7]. In the present case, we successfully covered the ruptured plaque and promptly trapped the large thrombus using a drug-eluting stent. Additionally, thrombolysis was achieved using dual antiplatelet agents plus a DOAC. Mechanical thrombectomy with a balloon can also be used in the management of thrombi [7]; however, this method is complicated when a massive thrombus in the coronary artery causes distal embolism and a slow flow or no-reflow phenomenon. Moreover, although distal embolic protection devices can effectively prevent distal embolism, they may lead to temporary or complete absence of coronary flow development. This leads to an unstable hemodynamic state necessitating a cardiac support device, such as an intra-aortic balloon pump or Impella device. Furthermore, due to their quantity, distal embolism protection devices cannot completely retrieve huge thrombi in the coronary artery. Therefore, thrombectomy or thrombolysis should be performed without distal embolism. Stenting for culprit lesions can also cause distal embolism; however, both stenting and nonstenting strategies can cause distal embolism during or after the procedure. In essence, the nonstenting strategy in the acute phase carries a risk of reocclusion of the culprit lesion in addition to distal embolism [7]. Thus, we considered an immediate stenting strategy for only ruptured plaques with trapping of the long and massive thrombus. Moreover, although a previous report has highlighted the usefulness of aspiration thrombectomy after proximal stenting [15], we did not perform it in this case. We evaluated that unsuccessful aspiration of the huge distal thrombi could worsen coronary flow and cause distal embolism. Hence, thrombolysis with antiplatelet agents plus DOAC seemed a more reasonable option and could avoid extended parenteral coagulation that inhibits cardiac rehabilitation in cases involving older adults [16].

A differed stenting strategy can be selected for managing patients with AMI during PCI, particularly if they have a massive thrombus and achieve TIMI Grade 2 or 3 flow [7]. However, these patients incur elevated healthcare costs and have the potential risk of reocclusion of the culprit lesion. Therefore, managing a massive thrombus is indeed challenging. However, if immediate stenting coupled with thrombolysis using a DOAC in dual antiplatelet therapy proves successful without distal embolism, this strategy could be considered a more effective treatment compared to the deferred stenting approach.

## 4. Limitation

In this case, we used antiplatelet agents plus DOAC instead of intravenous unfractionated heparin. Due to the patient's advanced age and susceptibility to delirium, we avoided performing continuous intravenous infusion of parenteral anticoagulants. However, in younger patients, this approach may not be appropriate. Considering the bleeding risk, particularly in younger patients with a low risk of delirium and intravenous drip self-removal, intravenous infusion such as unfractionated heparin should be considered instead of oral or enteral coagulants, such as DOAC.

While thrombolytic therapy with DOAC on dual antiplatelet agents successfully resolved the large intracoronary thrombi without causing distal embolism, large intracoronary thrombus still poses significant distal embolism risk. Therefore, depending on the case, the removal method of the residual large thrombus should be thoroughly explored.

In particular, when repeated aspiration thrombectomy fails to attain TIMI Grade 2 or 3 flow in younger patients, catheter-directed intracoronary thrombolysis using medications for large intracoronary thrombi may be warranted in treating AMI. Thus, if a large thrombus vanishes, the conventional immediate stenting strategy can manage the culprit AMI lesion. Furthermore, as we do not have sufficient evidence regarding whether this treatment regimen has the same effect among other older adults, more studies are needed to address the better strategy for coping with large thrombus in coronary arteries.

## 5. Conclusion

Massive thrombi are uncommon but not rare during PCI for AMI and constitute a significant factor contributing to the occurrence of slow flow or no-reflow phenomenon. We present a case of AMI with a large thrombus, where immediate stenting to cover the ruptured plaque, without encountering slow flow/no-reflow phenomenon, and employing thrombolysis through a combination of antiplatelet agents and a DOAC led to favorable procedural and clinical outcomes. This treatment strategy was useful for avoiding reocclusion of the culprit lesion and extended parenteral coagulation, as well as reducing healthcare costs in the present case.

## Data Availability

Data sharing is not applicable to this article as it is a case report.

## Nomenclature

AMI: acute myocardial infarction
CAG: coronary angiography
DOAC: direct oral anticoagulant
LAD: left anterior descending artery
PCI: percutaneous coronary intervention
TIMI: thrombolysis in myocardial infarction
